# The Role of Health Networks in Disseminating Knowledge about Infant HIV Testing in Rural Uganda: Population-Based Sociocentric Network Study

**DOI:** 10.1007/s10461-025-04873-x

**Published:** 2025-10-10

**Authors:** Alison B. Comfort, James Moody, Julian Adong, Carol S. Camlin, Theodore D. Ruel, Scholastic Ashaba, Jessica M. Perkins, Charles Baguma, Emily N. Satinsky, Justus Kananura, E. Betty Namara, Mercy Juliet, Bernard Kakuhikire, Cynthia C. Harper, Alexander C. Tsai

**Affiliations:** 1 Bixby Center for Global Reproductive Health, Department of Obstetrics, Gynecology and Reproductive Sciences, University of California San Francisco, San Francisco, CA, USA; 2 Department of Sociology, Duke University, Durham, NC, USA; 3 Mbarara University of Science and Technology, Mbarara, Uganda; 4 Division of Prevention Science, Department of Medicine, University of California San Francisco, San Francisco, CA, USA; 5 Division of Pediatric Infectious Disease and Global Health, Department of Pediatrics, University of California San Francisco, San Francisco, CA, USA; 6 Peabody College, Vanderbilt University, Nashville, TN, USA; 7 University of Southern California, Los Angeles, CA, USA; 8 Center for Global Health and Mongan Institute, Massachusetts General Hospital, Boston, MA, USA; 9 Harvard Medical School, Boston, MA, USA; 10 Harvard T.H. Chan School of Public Health, Boston, MA, USA

**Keywords:** Infant HIV testing, Infant diagnosis, HIV knowledge, HIV/AIDS, Social networks, Information dissemination, Uganda

## Abstract

Early testing of infants exposed to HIV can significantly decrease mortality for those linked to HIV treatment. Infants exposed to HIV should first be tested at 6 weeks of age, but only 60% are tested as recommended. Little research has focused on the role of social networks in disseminating information about infant HIV testing. We conducted a cross-sectional, sociocentric network study of all adults living in a rural parish in Uganda (N=1,383) and gathered data on socio-demographic characteristics, self-reported HIV status, and knowledge about infant testing recommendations. We administered a culturally-adapted name generator to measure the parish health network. We fitted a multivariable generalized linear regression model with a logit distribution to estimate the association between having at least one social tie with correct knowledge about early infant testing and individual knowledge about infant testing. Having at least one social tie who knew about infant HIV testing at 6 weeks was positively associated with correct knowledge about early infant testing (adjusted odds ratio [aOR] 1.42, 95% confidence interval [CI] 1.07- 1.88, p-value=0.02). Correct knowledge about early infant testing was also associated with having daily contact with social ties (aOR 1.31, 95% CI 1.00-1.71, p-value=0.05) and being considered an authority for health advice within the network (aOR 1.81, 95% CI 1.18-2.77, p-value=0.01). These findings suggest that interventions to enhance peer-to-peer information exchange could increase knowledge about early infant testing, since individuals rely on close, frequently contacted social ties. Network-central individuals can also be engaged to disseminate information about early infant testing

## Introduction

More than one million infants are exposed to HIV perinatally in high-burden African countries, leading to 120,000 new HIV infections among infants each year. Progress in reducing infant infections is slowing [1, 2]. HIV disease progression is also more rapid in infants than in adults, resulting in high rates of mortality in the first few months of life [3]. If untreated, half of infants with HIV will die by age 2 [4]. Early testing of infants exposed to HIV, with linkage to treatment for infants with HIV, can significantly decrease mortality [5, 6]. Yet, only 60% of infants exposed to HIV worldwide receive guideline-concordant testing at 6 weeks of age [7, 8]. While significant efforts have been made to scale-up access to early infant diagnosis, there remain important gaps in ensuring timely early infant diagnosis and treatment initiation and ultimately in reducing mortality among infants living with HIV [8, 9]. Without marked improvements in early infant diagnosis, the global community will not be able to achieve the 95-95-95 targets to end the AIDS epidemic by 2030 [10].

The World Health Organization (WHO) recommends that infants exposed to HIV be first tested at 6 weeks of age, with follow-up testing at 9 months and after breast-feeding ceases [11]. In contrast to adult HIV testing, infant testing requires the use of assays for nucleic acids (DNA or RNA) because testing for anti-HIV antibodies can result in false positives in children due to transplacental transfer of maternal antibodies which may persist into the second year of life [8, 12]. Given the challenges of offering nucleic acid testing (NAT) in resource-limited settings and requiring individuals to return for results, the WHO recently recommended point-of-care testing to speed results and initiation of treatment [8, 12-15]. Point-of-care DNA-PCR testing has increasingly become available, including in Uganda [12-15]. The recommendation for the first test to be conducted at 6 weeks, particularly in resource-limited settings, is based both on feasibility (i.e., coinciding with first vaccination visits for infants) and to capture any transmission that may have occurred late in pregnancy and during delivery due to the incubation period.

Maternal characteristics associated with delays in early infant testing include uncertainty about maternal serostatus [12, 16], non-disclosure to partner and others [17], older maternal age [18, 19], higher parity [20], and lower levels of education [17]. Lack of knowledge among mothers about prevention of transmission is also associated with delays in early infant testing [18, 19]. Much less research has focused on understanding whether mothers and caregivers of infants exposed to HIV know the recommended timing and frequency of early infant testing. One quantitative study among mothers living with HIV in Uganda found that only 20% knew the correct recommended timing and frequency of infant HIV testing; correct knowledge of timing was positively associated with uptake of infant testing [19]. In two other qualitative studies among mothers living with HIV in South Africa, few knew about recommended testing at 6 weeks of age, and this lack of knowledge contributed to not seeking early infant testing [18, 21]. Aside from these few studies among mothers living with HIV examining knowledge of recommended infant testing [18, 19, 21], limited research has focused on understanding community knowledge about early infant testing.

Social networks can serve as important sources of health information [22-24]. Social networks are broadly conceptualized as the social relationships that individuals have with each other [25]. Individuals tend to rely on their social networks for an array of health information. Several social network analyses have been conducted in African countries to examine the role of social networks on spreading health information including information on antenatal care in Uganda [26], contraception in Madagascar and Kenya [27, 28], and HIV risk perception in Kenya and Malawi [29, 30]. Other social network analyses conducted outside of the African context have studied topics including water purification [31], child nutrition [31], and COVID-19 [32]. Only a few examples exist within the African context using a sociocentric network approach (complete population within a bounded community). These studies specifically examined HIV-related behaviors. A sociocentric study in Tanzania found that HIV testing behaviors were significantly higher if men thought their network members had also tested [33], and peer encouragement was critical for decisions to seek HIV testing [34]. A recent sociocentric study in Kenya also found that engaging highly central nodes in the network resulted in significantly higher HIV testing, as well as linkage to services among those who tested [35].

Social networks can also be sources of misinformation about health topics [26, 30] and misperceptions about others’ engagement in health behaviors [36, 37]. Yet individuals often prefer turning to family members and friends for health information and advice rather than to health care providers [26, 27, 38]. Importantly, social networks can serve as sources and conduits of trusted information, especially for more stigmatizing health topics such as HIV [29, 30, 39]. Women living with HIV may prefer to rely on close social ties, such as their partner, mother, siblings, and friends, to access information related to HIV and their children [39-41]. To ensure that mothers and caregivers of infants exposed to HIV seek timely infant testing, it is important to understand whether the people in their social networks are aware of recommendations for infant HIV testing.

Individuals’ social networks will vary in their composition (e.g., number of ties to women, number of ties to other people living with HIV) and structure (e.g., density, centrality). Following social network theory, these differences in network characteristics will influence individuals’ exposure to health information as well as their ability to disseminate that knowledge [22-24]. Despite the important role that social networks can play in disseminating trusted, accurate information about recommended timing and frequency for early infant testing, there is limited research examining this question in general population samples within HIV-endemic settings. It is important to understand general knowledge about infant HIV testing and how it is disseminated through social networks to ensure timely uptake of infant HIV testing.

To address this gap in research about general population knowledge of infant HIV testing and the importance of social networks in spreading this information, we conducted a cross-sectional, sociocentric study of all adults living in an HIV-endemic, rural parish in Uganda to gather data on individuals’ health networks (i.e., the individuals they rely on for health information and advice). We surveyed study participants about their knowledge of recommended timing for early infant testing. We then combined these data with measures of health network composition and structure to identify which individuals tended to have correct knowledge about early infant testing. Our study sought to understand whether having social ties with correct knowledge of infant HIV testing was predictive of individuals’ own knowledge about early infant testing. We also sought to identify what network attributes characterized those with correct knowledge to inform strategies for identifying whom within networks are best placed to spread accurate, trusted information about infant HIV testing.

## Methods

### Study Setting and Participants

Study participants were recruited from 8 rural villages in Nyakabare Parish, Rwampara District, Uganda. These study participants have been enrolled in an ongoing longitudinal, sociocentric study that began in 2011 and which includes all adults 18 years or older (and emancipated minors aged 16–18 years) who consider Nyakabare Parish their primary residence. People who cannot communicate effectively with study staff (e.g., due to deafness, acute intoxication) are excluded. The study follows an open cohort design: at each wave, new study participants are included if they either aged into the study or migrated into the study catchment area. The analysis described in this manuscript used data from the 2021-22 survey wave, when survey questions on infant HIV testing were first added. A total of 1,566 individuals participated in that survey wave, with a response rate of 91%. Due to an administrative delay, the survey module on infant HIV testing was not added until after 183 study participants had already been surveyed. Thus, the analytic sample for the present study included 1,383 participants.

### Study Procedures and Data Collection

Once study participants were recruited and consented, we collected survey data on socio-demographic characteristics and self-reported HIV status. We also administered a culturally-adapted name generator eliciting participants’ health networks, to which study participants could nominate up to 6 other adults in the study parish: *“Over the past 12 months*, *with whom in this parish have you usually discussed any kind of health issue? Examples of health issues might include topics like your child’s health*, *family planning*, *nutrition*, *HIV*, *mental health*, *immunizations*, *sanitation methods*, *alcohol abuse or other issues.”* This name generator was adapted to elicit health networks within the local context by including behaviorally-specific prompts, with examples of health-related matters that our qualitative data and key informant interviews indicated were common topics of discussion in the local context (e.g. immunizations, HIV).

In network analysis, the survey respondent (labeled the “*ego*”) nominates individuals (termed the “*alters*”). The study’s sociocentric design meant that it captured a full census of all individuals within a bounded geographic area and the social relationships that existed between the individuals (nodes) [23]. All adults across Nyakabare Parish were surveyed and served as egos in the study. The sociocentric design meant that an ego could also serve as an alter. When egos nominated alters (from within the study parish) in their health network, this nomination constituted a social relation (i.e., *social tie)* within their health network. Our study’s focus was on health nominations, and nominations were capped at 6 alters. Median nominations were 2 individuals, with the interquartile range going from 1 to 3, and the mean equal to 1.8. This distribution suggested that the bound on 6 nominations did not limit nominations meaningfully and that we captured individuals’ full health network. Only 8 individuals (< 1%) nominated 6 people.

In addition to health network data, we administered a survey module on knowledge about infant HIV testing where we elicited study participant beliefs about the risk of HIV transmission from mothers to infants and knowledge about testing recommendations for infants exposed to HIV. All survey questions were written in English, translated to Runyankole, pilot-tested for comprehension, revised as needed, and back-translated to verify fidelity to the original English.

Data were initially collected in-person, typically at study participants’ homes. Once restrictions on internal movement were imposed during the COVID-19 pandemic, and in accordance with Uganda National Council for Science and Technology guidelines on the conduct of research during the pandemic, all remaining study recruitment, consenting, and data collection were converted to telephone-based procedures. Trained data collectors administered the surveys in Runyankole using the Computer Assisted Survey Information Collection (CASIC) Builder^™^ software (West Portal Software Corporation, San Francisco, CA, USA) programmed into study laptops. To ensure accuracy in identification of nominated alters, the software was programmed with an automated look-up feature which allowed the participant to view the alter’s photograph to confirm their nomination. Study participants provided consent separately to participate in the survey and to have their photo taken. One study participant consented to participating in the survey but opted out of having their photograph taken.

### Study Measures

Our survey questions asked study participants about the risk of HIV transmission from mothers to infants following pre-validated questions from the Demographic and Health Surveys [42, 43]. In separate questions, study participants were asked if HIV could be transmitted from a mother to her baby during pregnancy, during delivery, and by breastfeeding, with “yes”, “no”, and “don’t know/unsure” as possible responses. They were then asked about infant HIV testing using the following question: *“When a mother living with HIV gives birth to her baby*, *how many times should that baby be tested for HIV?”* with responses including “babies do not need to be tested for HIV”, “once”, “twice”, “three times”, “more than three times”, or “don’t know/unsure”. Those who believed babies exposed to HIV should be tested at least once were then asked follow-up questions: “*At what age(s) should that baby be tested for HIV?”* with prompts for “*at 6 weeks”*, *“at 9 months”*, and *“after the mother stops breastfeeding”*, *with “*yes”, “no”, and “don’t know/unsure” as possible responses. Our study team developed and piloted these questions because there were no pre-validated infant testing knowledge questions in the literature.

The primary outcome of interest was a binary indicator denoting whether study participants knew that an infant exposed to HIV should be tested for HIV at 6 weeks. For the primary exposures of interest, we constructed measures for network structure and network composition. We labeled individuals as having no health network if they nominated no one in response to the health network name generator (although they could be nominated by others). We defined complete social isolation for individuals who nominated no one in response to the health network name generator (outdegree = 0) and who also were not nominated by any other study participant (indegree = 0).

We generated several network structure measures among the non-isolated individuals. Centrality measures included: indegree – the number of incoming ties from alters seeking health information and advice from the ego, and outdegree – the number of alters the ego nominated from whom the ego sought health information and advice. We also included eigenvector centrality, representing the number of alters weighted by the alters’ centrality. We examined closeness centrality, using harmonic centrality which captured the average distance to all other nodes (where a first-degree tie had a distance of 1 unit, a second-degree tie had a distance of 2 units, etc.). Harmonic centrality was inversed and normalized such that larger values reflected greater centrality (meaning egos had fewer “degrees of freedom” linking them to others in the network). We included a measure of authority, which captured egos with an especially high number of incoming ties relative to others in the network, and a measure for hub, which captured egos with significantly higher numbers of social ties (incoming and outgoing) compared to other nodes. We also measured constraint, the extent to which a person’s network was concentrated with redundant contacts and less evenly distributed (i.e., “all my friends know each other”) [44]. All network structure measures were defined at the level of the parish rather than at the level of the village because there was a substantial number of inter-village nominations (ranging from 7 to 18% of total nominations by village).

Given our sociocentric (whole population) design, each study participant could serve both as an ego and an alter, and their self-reported characteristics could be used to generate network composition measures. Network composition measures were defined for the sample of non-isolated individuals. We included a continuous variable for the number of alters who had correct knowledge about recommended infant HIV testing at 6 weeks and a related binary measure for having at least one alter with correct knowledge about infant HIV testing at 6 weeks. We also included a binary measure for individuals with high levels of trust in the network (greater than or equal to median trust in sample, where trust was rated on a scale of 1 [low trust] to 10 [high trust] and averaged across all alters) and a binary measure for individuals who saw many alters on a daily basis (greater than or equal to median number of alters seen every day). Other binary composition measures included: having at least one female in the network, having at least one health network member living with HIV, having at least one friend in the network, having at least one alter with some primary education or more, and having at least one alter who had children. We also generated a measure for mean age of alters.

### Ego-level Characteristics

We collected data on sociodemographic characteristics and included them in the regression models as ego-level variables: age, female sex, educational attainment (none, some primary school, completed primary school, and completed more than primary school), self-reported HIV status, marital status (married/cohabitating, separated/divorced/widowed, or single/never married), number of biological children still alive, and household asset wealth (categorized in quintiles ranging from poorest to least poor) [45]. In the presentation of descriptive statistics, we described separately land ownership, livestock ownership, presence of a flush toilet in the home, and number of rooms in the home. These were not included in the regression models since their values were reflected in the household asset wealth measure. Missingness was minimal on ego characteristics, except for age (1% of sample missing), number of children (6% missing), and self-reported HIV status (5% missing).

### Statistical Analysis

First, we calculated summary statistics of the study population, including network structure and composition measures (Table 1). We then fitted multivariable generalized linear regression models with a logit distribution, adjusting for ego-level characteristics and using cluster-correlated robust standard errors to account for within-village similarities. All analyses included research assistant fixed effects and village fixed effects [46-49]. Exponentiated regression coefficients were interpreted as adjusted odds ratios. Missingness on age, number of children, and self-reported HIV status would have resulted in exclusion of 150 observations (11% of analytic sample) from the primary multivariable regression models. To avoid dropping observations due to missingness as described above, we used dummy variable imputation for these variables [50]. For the categorical variable (self-reported HIV status), we created a separate category taking a value of 2 if missing; for continuous variables (age and number of children), we replaced missing values with a number out of range and included a dummy variable (both for age and number of children) that took on a value of 1 if that value was missing and 0 if it was not missing, so that it would be treated separately in the model.

We presented summary statistics for our HIV knowlege outcomes (Table 2). We then explored differences in ego-level characteristics between those who were isolated and those who were not isolated with χ^2^ tests and t-tests (Table 7-Appendix). Our main set of analyses (Table 3) showed the full model, with our primary outcome for knowing correct timing of infant testing at 6 weeks, including ego-level covariates and the network predictor (i.e., whether ego had at least 1 alter with correct knowledge about timing of infant HIV testing). For subsequent models (Table 4), we reported only the coefficient representing network structure and composition while not displaying ego-level characteristics. Certain network structure measures were highly skewed in the sample (eigenvector centrality, authority score, and hub score; skewness = 10.65, 35.25, 6.85 respectively), so we defined them instead as binary measures to capture those in the top 10th percentile of the sample. Since harmonic centrality was left-skewed (skewness = −2.43), we defined a binary variable identifying those not in the lowest 10th percentile so that all centrality measures could be interpreted in a similar direction. We assessed the association between our main outcome and being isolated and having no health network, respectively (Table 5). We then explored differences in characteristics for network structure measures that were predictive of our outcome. We compared both ego self-reported characteristics and characteristics reported when these individuals were nominated as alters (e.g., level of trust), averaged across all instances of nomination (Table 6).

### Ethics

All study materials were approved by the Mbarara University of Science and Technology Research Ethics Committee and the Mass General Brigham Human Research Office, with research reliance agreements with the University of California at San Francisco. Consistent with national guidelines, we obtained clearance to conduct the study from the Uganda National Council for Science and Technology and the President’s Office. Study participants provided written informed consent. If they were unable to write, they were permitted to indicate consent with a thumbprint in the presence of a witness. When study procedures were converted to telephone interviews, study participants provided verbal consent that was documented by the interviewing research assistant. Consistent with local custom, study participants received either a kilogram of sugar or a bar of soap as a study participation incentive.

## Results

### Summary Characteristics

**Fig. 1:**
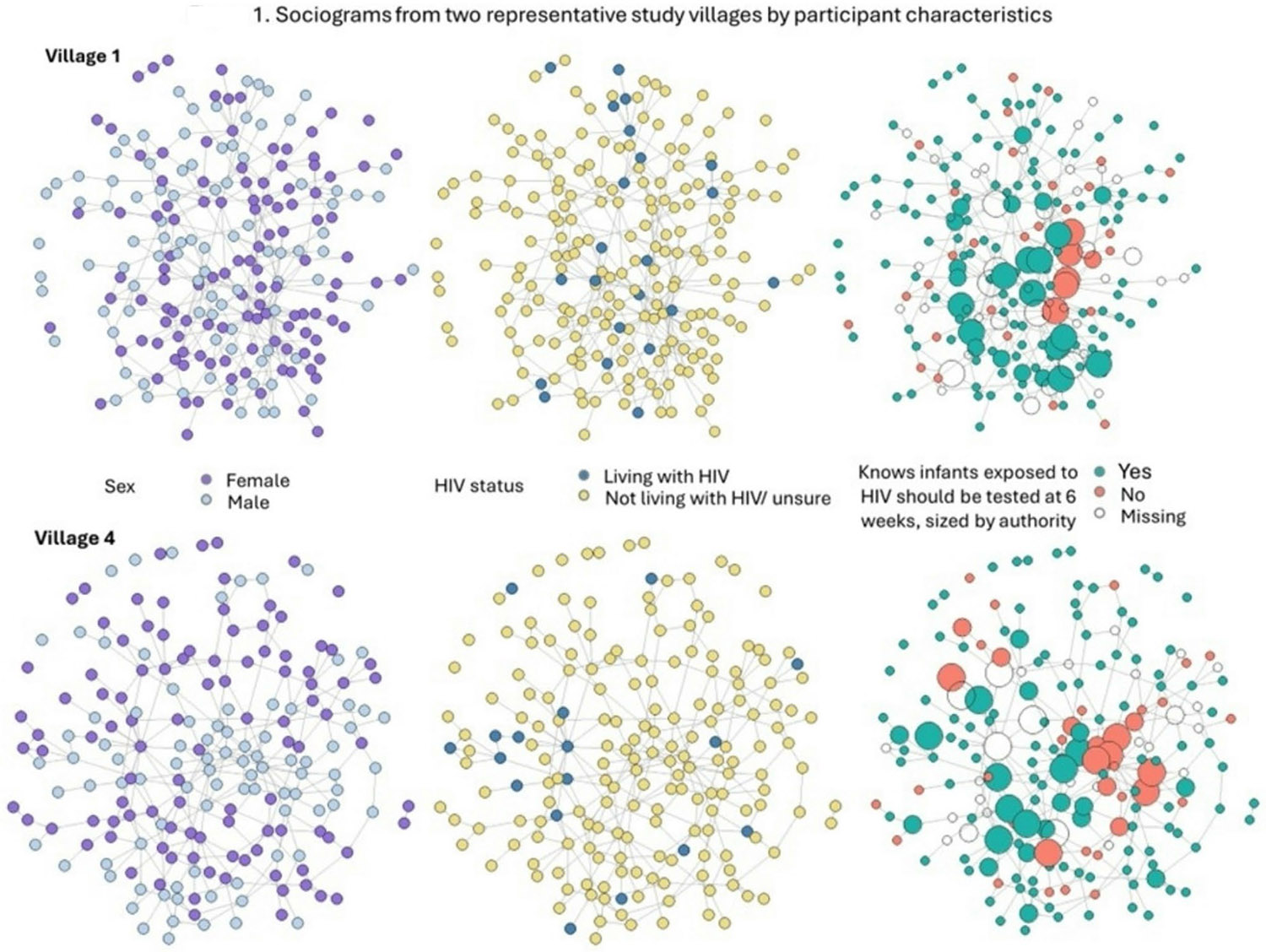
Sociograms from two representative study villages by participant characteristics

Roughly half of study participants were female (53%). Their median age was 40 years old (interquartile range [IQR], 29–53). Two-thirds of the participants were either married or cohabitating (64%), and 11% (*n* = 152) self-reported living with HIV. Remaining sample characteristics are reported in Table 1. Most participants were aware that infants exposed to HIV should be tested at 6 weeks (78%; Table 2) while 19% responded that they did not know and 3% responded no. Figure 1 shows the sociograms for two of the study villages with differences by sex, self-reported HIV status, and knowledge of infant testing at 6 weeks where nodes were sized by authority score.

Almost one-tenth (7%) of the sample was isolated with no incoming or outgoing ties. When we compared ego-level characteristics between individuals who were isolated versus non-isolated, we found that women were less likely to be isolated (43% vs. 56%, p-value = 0.005). Individuals who were single or never married were more likely to be isolated (34% vs. 15%, p-value = 0.000). Individuals who were isolated were less likely to have no education (2% vs. 13%) and more likely to have more than primary schooling (56% vs. 37%, p-value = 0.000). Individuals who were isolated were also younger (mean of 35 years old vs. 43 years old, p-value = 0.000). Other characteristics (serostatus and number of children) were not significantly different between the two groups.

Among the non-isolates, 10% had no health network meaning they had no one they sought health advice or information from (though they had incoming ties). When we included isolated individuals, we found that two-thirds had at least one alter in their health network who knew that infants exposed to HIV should be tested at 6 weeks of age (66%), meaning that one-third of individuals had no one in their health network with correct knowledge about infant HIV testing. When isolated individuals were excluded, the proportion with at least one alter who knew about infant HIV testing at 6 weeks rose to 78%. In terms of distribution, about one-half of non-isolates had only 1 alter in their health network who knew about infant HIV testing (45%), one-fifth had 2 alters who knew this, and 11% had 3 or more alters who had this knowledge.

Among non-isolates, mean indegree was 1.7 and mean outdegree was 1.8, meaning individuals on average had 2 alters they relied on for health information and advice and 2 individuals relied on them for health advice and information. Two-thirds of individuals reported seeing 2 or more alters every day. Almost one-third had no women in their health network (29%) and three-quarters had no one living with HIV in their network (75%). One-fifth had one alter in their network who was living with HIV, while 2% had 2 or more alters in their network who were living with HIV. One-third had no friends in their health network (as opposed to family members and other social ties). Average trust in alters was high (8 on a scale from 1 to 10, with 10 representing complete trust).

### Association between Alter Knowledge of Infant Testing and Ego Knowledge of Infant HIV Testing at 6 Weeks

In our multivariable regression models (Table 3), correct knowledge among egos about the need for infant HIV testing at 6 weeks was positively associated with having at least one alter who knew about infant HIV testing at 6 weeks (aOR 1.42, 95% CI 1.07–1.88, p-value = 0.02). Post-estimation predicted margins showed that 79% of individuals had correct knowledge if they had one or more alters with correct knowledge, compared to 74% if they had no alters with correct knowledge, representing a 5-percentage point increase in the probability of correct knowledge about early infant testing.

There appeared to be a threshold effect between having alters with knowledge about infant testing at 6 weeks and ego’s own knowledge about infant testing (Table 4). While having at least one alter with this knowledge was predictive of ego’s own knowledge about infant testing, there was no linear relationship between the number of alters in their health network with knowledge of infant testing and ego’s own knowledge.

Several ego-level characteristics were also associated with correct knowledge of infant HIV testing at 6 weeks (Table 3). Women were more likely to report knowing about the need for infant HIV testing at 6 weeks (aOR 2.31, 95% CI 1.59–3.37, p-value < 0.001), as were individuals who had completed primary school (aOR 1.79, 95% CI 1.04–3.10, p-value = 0.04) or had more than a primary school education (aOR 1.70, 95% CI 1.09–2.65, p-value = 0.02). Individuals who were single or never married were less likely to report knowing about infant HIV testing at 6 weeks (aOR 0.58, 95% CI 0.44–0.76; p-value < 0.001). Age was negatively associated with knowing about infant HIV testing at 6 weeks (aOR 0.97, 95% CI 0.96–0.98, p-value < 0.01). The association between self-reported HIV serostatus and knowledge of infant HIV testing at 6 weeks was not statistically significant (aOR 1.72, 95% CI 0.82–3.61, p-value = 0.15).

### Association between Other Network Composition and Network Structure Measures and Ego Knowledge of Infant HIV Testing at 6 Weeks

Having daily contact with a high number of alters was positively predictive of ego’s own knowledge about infant testing (Table 4). Those who reported daily contact with a high number of alters (greater than or equal to median number of alters with daily contact) had 1.3 higher odds of having correct knowledge about infant HIV testing (aOR 1.31, 95% CI 1.00-1.71, p-value = 0.05). Other measures of network composition were not predictive of ego’s knowledge about infant HIV testing, including having at least one female in the health network, having at least one person living with HIV in the health network, having at least one alter with some primary education or more, and having at least one alter with children in the health network.

Having higher authority scores was positively predictive of having correct knowledge of infant HIV testing. Individuals who were in the top 10th percentile for authority score had 1.8 higher odds of having correct knowledge about infant HIV testing at 6 weeks (aOR 1.81, 95% CI 1.18–2.77, p-value = 0.01). There was suggestive evidence of a positive association between having a high hub score and having correct knowledge of infant HIV testing at 6 weeks (aOR 1.25, 95% CI 0.98–1.59, p-value = 0.07), although it did not reach significance. Other measures of network composition, including indegree, outdegree, harmonic centrality (closeness), and eigenvector centrality were not predictive of having correct knowledge of infant HIV testing at 6 weeks.

### Association between Isolation and Ego Knowledge of Infant HIV Testing at 6 Weeks

Our next analyses included isolated individuals in the sample, meaning those with no incoming or outgoing health network ties (Table 5). We found that being isolated was not predictive of knowledge of infant HIV testing, nor was having no health network.

### Characteristics of Individuals with High Authority Scores Compared to Others

There were several differences in characteristics between those with high authority scores (top 10th percentile) and all other nominated individuals (Table 6). Those with high authority were more likely to be married (78% vs. 64%, χ^2^ = 20.74, p-value = 0.000). On average, they were older by 10 years (t-stat = 6.50, p-value = 0.000). They had significantly higher indegree (4.4 vs. 1.4, χ^2^ = 16.54, p-value = 0.000). There was no difference between those with high versus low authority scores in being considered highly trusted by those who nominated them. However, those with high authority scores were less likely to be seen daily (46% vs. 57%, χ^2^ = 17.30, p-value = 0.000) and were less likely to be the ego’s spouse/partner (5% vs. 10%, χ^2^ = 12.94, p-value = 0.000). They were also less likely to be nominated first (as a person’s most important health tie; 11% vs. 19%, χ^2^ = 16.42, p-value = 0.000), and they were more likely to be nominated third (23% vs. 18%, χ^2^ = 5.02, p-value = 0.025). Individuals with high authority scores did not differ by gender, education level, self-reported HIV status, or number of children.

## Discussion

In this population-based sociocentric social network study in rural Uganda, we explored which individuals had correct knowledge about timing of infant HIV testing at 6 weeks and what social network characteristics predicted correct knowledge about early infant testing. We found that individuals who had at least one social tie with correct knowledge about timing of early infant HIV testing were more likely to also have correct knowledge about early infant testing. There was no apparent linear relationship between the number of alters with correct knowledge about early infant testing and egos’ own knowledge. Together, these findings are suggestive of a local contagion effect through direct ties to someone with correct knowledge. There also appeared to be a threshold effect, meaning that individuals needed to have just one social tie in their network with correct knowledge about early infant testing to be more likely to have correct knowledge. This finding is consistent with simple contagion models of information dissemination in networks, which predict that only one social tie is needed for information dissemination [51]. These models contrast with complex contagion models for behavior change which require more than one alter to influence changes in outcomes [51].

While our findings are consistent with contagion, through social influence from peer effects (i.e., individuals learning from others in their health network), there is also the possibility that these results are due to homophily, meaning the tendency for individuals to associate with others who are similar [52]. We cannot rule out homophily given the cross-sectional nature of these data. Furthermore, if the name generator had specifically focused on discussions about infant HIV testing, other social ties may have been included. Finally, our alter measures represent self-reported knowledge by those individuals rather than the ego’s perceptions of alters’ knowledge, which may differ and result in over-estimating or under-estimating alters’ knowledge as shown in other studies [36, 53, 54]. Other study designs are needed to be able to separate the extent to which these associations are attributed to contagion/social influence versus homophily.

Certain ego-level characteristics were predictive of knowledge about infant testing. Women, those with more education, and those who were younger were more likely to have correct knowledge, while those who were single were less likely to have correct knowledge. Interestingly, many alter characteristics (i.e., sex, HIV serostatus, education level, number of children) were not predictive of egos’ knowledge of early infant testing. Instead, we observed a relationship between having a high number of frequently-contacted alters and having correct knowledge of early infant testing. The absence of a relationship between other alter characteristics and egos’ knowledge may have been because these nominated alters represented highly trusted individuals whom individuals saw relatively often and whom egos had purposely selected for seeking out health information and advice. The strength of these ties may be a more important feature of the health network’s influence compared to other alter attributes (such as sex, age, education level, serostatus) in determining from whom individuals decided to seek health information and advice. Relying on frequently contacted social ties, who are likely close and highly trusted, for health information is consistent with findings from other studies and is important for stigmatizing health topics such as HIV [26, 27, 29, 30, 38, 39]. A population-based study in Malawi, which found a positive association between social network members’ perceptions of HIV risk and individuals’ own perception of HIV risk, confirmed the importance of individuals’ social networks for learning HIV-related information [29, 30]. Other studies have also shown that relying on close trusted social ties to obtain health information and support is especially important for people living with HIV [39-41]. We need to ensure that close social ties share knowledge with each other, regardless of ties’ particular attributes. This approach builds on examples using trusted messengers to convey information about health issues including vaccine hesitancy and other health topics [55, 56]. Health education campaigns therefore need to include messaging on the importance of discussing potentially stigmatizing health topics with close social relations.

We also found that certain network structure characteristics were associated with correct knowledge of early infant testing, meaning that there was a network position effect. Individuals with high authority scores were more likely to have correct knowledge of early infant testing. Authorities in a network are nodes with an especially high number of incoming ties. Many individuals tend to seek information from authorities because they represent knowledgeable, trustworthy information sources within the network [57]. Those with high authority scores may occupy central positions in a network due to a visibility effect related to having correct knowledge—others in the community may be able to identify who is most likely to be an authority on accurate health information and seek them out for this information. Our exploratory analyses found that those who were older and married/partnered were more likely to be considered authorities; interestingly, they did not tend to be more highly trusted by those who nominated them, and they were not seen as frequently. Authorities also tended to be chosen later, in terms of importance, in the list of nominations. These results suggest that those who are considered authorities may be considered weak ties, as opposed to highly trusted, frequently seen ties [58]. These findings are consistent with research by Small highlighting the importance of weak ties in sharing important information [59]. Yet these individuals are viewed as important sources within health networks for correct information about infant HIV testing and should be identified and engaged to help spread this information.

There was also suggestive evidence that those with high hub scores (i.e., those with an especially high number of both incoming and outgoing ties) were more likely to have correct knowledge. In general, hubs tend to know who the authorities are in the network and are likely to link to them. Yet other centrality measures (indegree, eigenvector centrality, harmonic centrality) did not predict correct knowledge about early infant testing. Overall, these findings show that there was some role in the network for network position, meaning that those who were highly sought after for health advice and information did in fact have correct knowledge, though not all centrality metrics were predictive of correct knowledge. Because it is important to have at least one direct tie to someone who knows about early infant testing, it would be helpful to identify who tends to be viewed as authorities in the network in order to encourage them to share and effectively disseminate accurate information in the network.

Finally, when we included isolates in the analytic sample, we did not find an association between having no health network and knowledge of early infant testing. It is not possible to compare what would occur if individuals who were isolated had at least one social tie with correct knowledge about early infant testing. Our results showed that men and younger and more educated individuals were more likely to be isolated. These findings suggest that, at least for this health topic, not having a health network may not be detrimental for knowledge about infant HIV testing. Instead, these particular individuals may tend to rely on other information sources outside of their health network (e.g., media) which does not negatively affect this particular knowledge outcome. Isolation as we have defined represents information seeking tendencies, and includes individuals who do not seek out health advice from others in their community; it therefore does not represent social isolation which has been shown to be detrimental to health outcomes [60].

Our findings have implications for the design of interventions that ensure broad population knowledge about timing of early infant testing. Seeding information within a network among highly connected individuals is a commonly proposed strategy for ensuring broad information dissemination [29, 61]. Seeding information among individuals with high closeness (i.e., harmonic centrality) is effective because they reach many individuals directly [23]. However, our findings demonstrated that those with high closeness did not necessarily have correct knowledge and were not viewed as authorities within the community. It is therefore important to identify and encourage individuals viewed as authorities to disseminate information about early infant testing because they already occupy a recognized role in the community vis-à-vis being sought after for health information and having correct knowledge. Even if they do not represent strong ties within their health networks, their weak ties nonetheless serve an important purpose in spreading correct information. Secondly, it is important to identify and educate those with high closeness because, while they are not distinguished as having correct knowledge, they can reach many individuals. By virtue of our data, we could identify those more likely to serve as authorities (women, older individuals, more educated individuals, and those with higher socio-economic status). However, practical ways of identifying those individuals, due to the infeasibility of implementing fully mapped networks, could rely on asking community members to report who would be most effective in disseminating health information, consistent with approaches by Banerjee, Chandrasekhar, Duflo, Jackson [62].

This study has several limitations. First, our identification of an association between alter knowledge of infant testing and ego knowledge of infant testing cannot be entirely attributed to contagion or social influence. Individuals are likely to be clustered in health networks with individuals who are similar to themselves (*homophily).* Our cross-sectional study design cannot separate these social influence effects from homophily. Our name generator focused broadly on health topics, so we cannot ascertain that alters communicated this specific information. We also do not have information on ego’s perceptions of alters’ knowledge, which may differ (over/under-estimate) from their actual knowledge. Other study designs could overcome these limitations and establish the role of contagion/social influence on knowledge of infant testing. Second, the outcome measures may be over-estimated because of the way they were asked, with yes, no and don’t know as response options. Because there were no pre-existing established metrics for measuring knowledge of early infant testing, we designed and pilot-tested the questions ourselves, following a similar format as questions about maternal-to-child transmission used in the Demographic and Health Surveys [42]. Due to potential social desirability bias, study participants could conceivably have answered yes even if they did not know the recommended timing for the first HIV test for infants exposed to HIV. Since our predictor variable (i.e., having at least one alter with knowledge about early infant testing) would also suffer from the same potential bias in the same direction, this bias was not expected to affect the direction, magnitude, and/or statistical significance of this association. Third, there may also be differences in how data collectors administered this question. The data showed that, for one data collector, there were significantly more instances of positive responses. If we excluded observations from this data collector, the estimated association became larger in magnitude and more precisely estimated (aOR = 1.45 vs. 1.41 and p-value = 0.004 vs. 0.016). Fourth, collecting social network data via name generators typically entails concerns related to under-reporting of social network ties, both due to interviewer effects and study participant effects [46-49]. If egos are not presented with a roster of potential alters, they could forget to nominate certain social ties [63]. Our regression models included data collector fixed effects to mitigate these potential concerns. Feedback from the research team confirmed that they believed study participants reported their social networks with accuracy, and that participants were accustomed to the surveys and enjoyed discussing their social networks.

## Conclusion

It is critical for parents and caregivers of infants exposed to HIV to know the recommended timing of early infant testing to identify and promptly initiate treatment for infants living with HIV. Health networks act as important sources of health information and advice, especially for potentially stigmatizing topics such as HIV in children. Our findings demonstrated that having at least one individual in one’s health network who knew about recommended timing of early infant testing was critical to ensuring individuals knew these recommendations and suggestive that information-sharing within networks was occuring through social influence. Interventions to educate about early infant testing need to focus on the importance of peer-to-peer exchange of information since close, trusted social ties are those whom individuals rely on for this information. There is also an opportunity to target highly connected individuals in communities, specifically those viewed as leaders with correct information, to ensure they have this information and are encouraged to share it directly with those who rely on them for health information and advice.

## Figures and Tables

**Table 1:** Summary characteristics of study sample

Variable	*N* = 1,383^1^
*Sex*	
Female	739 (53%)
Male	643 (47%)
*HIV status*	
Living with HIV	152 (11%)
Not living with HIV / Unsure	1,179 (89%)
*Marital status*	
Married/cohabitating	891 (64%)
Separated/divorced/widowed	248 (18%)
Single/never married	243 (18%)
*Educational attainment*	
None	164 (12%)
Some primary (P1-P6)	390 (28%)
Completed primary (P7-P8)	289 (21%)
More than primary (S1-S6, vocational, university)	539 (39%)
Age	40 (29, 53)
Asset index rel. to parish (higher = wealthier)	−0.57 (−1.33,0.74)
Biological children still alive	3 (2, 5)
Land ownership	1,314 (95%)
Livestock ownership	780 (56%)
Number of rooms	4 (3, 5)
Ownership of flush toilet	
Has flush toilet	103 (8%)
Does not have flush toilet	1,279 (93%)

1 Median (IQR) or Frequency (%). Missingness for age was 1%, missingness for number of children was 6% and missingness for self-reported HIV status was 5%.

**Table 2:** HIV knowledge measures and health network characteristics

Variable	*N* = 1,383^1^
Ego knows that infants exposed to HIV should be tested at 6 weeks	
Yes	1,077 (78%)
No	45 (3%)
Don’t know	261 (19%)
*Network composition*	
Ego has at least one alter who knows about infant HIV testing at 6 weeks (including isolated individuals)	917 (66%)
*Excluding isolated individuals*	
Ego has at least one alter who knows about infant HIV testing at 6 weeks	917 (78%)
Number of alters who know about infant HIV testing at 6 weeks	
0	252 (22%)
1	527 (45%)
2	254 (22%)
3	107 (9%)
4	25 (2%)
5	4 (0.3%)
Average level of trust in alters from 1 to 10	8.4 (1.51)
Number of alters whom ego sees everyday	
1	414 (35%)
2	428 (37%)
3	200 (17%)
4	90 (8%)
5	29 (3%)
6	8 (0.7%)
Number of women in health network	
0	336 (29%)
1	474 (41%)
2	211 (18%)
3	103 (9%)
4	32 (9%)
5	12 (1%)
6	1 (< 0.1%)
Number of people living with HIV in health network	
0	881 (75%)
1	249 (21%)
2	37 (3%)
3	2 (0.2%)
Number of friends in health network	
0	361 (31%)
1	400 (34%)
2	237 (20%)
3	117 (10%)
4	41 (4%)
5	11 (1%)
6	2 (0.2%)
Number of alters who completed some primary school or more	
0	83 (6%)
1	585 (44%)
2	377 (28%)
3	192 (15%)
4	66 (5%)
5+	21 (2%)
Mean number of children among alters	4.5 (2.14)
Mean age of alters	48 (12)
*Network structure*	
Isolated (no incoming or outgoing health ties)	100 (7%)
*Among non-isolates*	
No health advice network (no outgoing health ties)	114 (9%)
Indegree	1.67 (2.11)
Outdegree	1.82 (1.19)
Closeness (harmonic centrality)	0.15 (0.03)
Eigenvector centrality	0.014 (0.051)
Authority	0.001 (0.006)
Hub	0.018 (0.116)
Constraint	0.50 (0.29)

1 Mean (standard deviation) or Frequency (%)

**Table 3:** Correlates of ego knowledge of infant HIV testing at 6 weeks and alter knowledge of infant HIV testing

	aOR	95% CI	*p*-value
At least one alter knows about infant HIV testing at 6 weeks	1.42*	[1.07–1.88]	0.02
Female	2.31***	[1.59–3.37]	0.00
Living with HIV (self-reported)	1.72	[0.82–3.61]	0.15
Married/cohabitating (*reference group)*	-	-	-
Separated/ divorced/ widowed	0.76	[0.51–1.12]	0.17
Single/never married	0.58***	[0.44–0.76]	0.00
No education *(reference group)*	-	-	-
Some primary school	1.30	[0.94–1.79]	0.12
Completed primary school	1.79*	[1.04–3.10]	0.04
More than primary school	1.70*	[1.09–2.65]	0.02
Age (years)	0.97***	[0.96–0.98]	0.00
Children (n)	1.01	[0.94–1.09]	0.78
Asset index Observations	1.01	[0.93–1.10]	0.86
Observations	1,169		

Table displays regression model results from generalized linear model with exponentiated coefficients for adjusted odds ratios. Models use cluster robust standard errors clustered by village. Covariate adjustment includes village fixed effects and research assistant effects and dummy variables for missing data imputation for age, number of children, and self-reported HIV status. * *p* < 0.05; ** *p* < 0.01; *** *p* < 0. 001; aOR = adjusted odds ratio; CI = confidence interval

**Table 4:** Correlates of ego knowledge of infant HIV testing at 6 weeks and health network composition and structure (among non-isolated individuals)

	*Outcome*: Ego knows about infant HIV testing at weeks of age		
	aOR	95% CI	p-value
***Health network composition***			
Number of alters who know about infant HIV testing at 6 weeks (n)	0.99	[0.90–1.09]	0.85
High trust in network (0/1)	1.03	[0.78–1.37]	0.84
Daily contact with many alters (0/1)	1.31*	[1.00–1.71]	0.05
At least one female in network (0/1)	1.14	[0.82–1.58]	0.45
At least one PLHIV in network (0/1)	1.01	[0.69–1.49]	0.94
At least one friend in network (0/1)	1.22	[0.91–1.62]	0.18
At least one alter with some primary education or more (0/1)	0.75	[0.50–1.13]	0.17
At least one alter with children in network (0/1)	1.13	[0.72–1.76]	0.59
Mean age of alters (n)	1.01	[0.99–1.03]	0.31
***Health network structure***			
Indegree	0.98	[0.91–1.05]	0.51
Outdegree	0.97	[0.87–1.09]	0.65
High closeness (harmonic centrality) (0/1)	1.69	[0.86–3.34]	0.13
High eigenvector centrality (0/1)	0.90	[0.45–1.80]	0.77
High authority (0/1)	1.81**	[1.18–2.77]	0.01
High hub (0/1)	1.25^	[0.98–1.59]	0.07
Constraint	0.84	[0.49–1.44]	0.54

Each row represents results from separate generalized linear models with exponentiated coefficients for adjusted odds ratios. Models use cluster robust standard errors clustered by village. Covariate adjustment includes ego sex, self-reported HIV status, age, marital status, educational level, asset index, biological children alive, village fixed effects and research assistant fixed effects. Trust measure is binary identifying those with average trust in alters greater than or equal to median trust in network. Frequency of contact is binary identifying those who see a high number of alters daily, great than or equal to median number of alters seen daily. Other composition measures are also binary identify at least one female, at least one PLHIV, at least one friend, and at least one alter with children. Measures for eigenvector centrality, authority and hub are binary measures identifying observations in the top 10th percentile. Closeness (harmonic centrality) is also a binary measure identifying observations above the lowest 10th percentile (given left skewness of data). ^ *p* < 0.10, * *p* < 0.05, ** *p* < 0.01,*** *p* < 0.001; aOR = Adjusted Odds Ratio, CI = Confidence Interval, PLHIV = people living with HIV

**Table 5:** Correlates of ego knowledge of infant HIV testing t 6 weeks and being isolated

	*Outcome*: Ego knows about infant HIV testing at by weeks of age		
	aOR	95% CI	p-value
Isolated (0/1)	0.79	[0.49–1.27]	0.33
No health advice network (0/1)	0.86	[0.56–1.31]	0.48

Each row represents results from separate generalized linear models with exponentiated coefficients for adjusted odds ratios. Models use cluster robust standard errors clustered by village. Covariate adjustment includes ego sex, self-reported HIV status, age, marital status, educational level, asset index, biological children alive, village fixed effects and research assistant fixed effects. Analytic sample includes individuals who are isolated (zero indegree and zero outdegree) and those with no health network (zero outdegree). ^ *p* < 0.10, * *p* < 0.05, ** *p* < 0.01, *** *p* < 0.001; aOR = Adjusted Odds Ratio, CI = Confidence Interval

**Table 6:** Differences in characteristics between individuals with high authority scores versus others in network

	Authorities within network	Not authorities within network	Χ^2^ / t-stat	*p*-value
*Ego level self-reported characteristics*				
Female	60%	54%	1.49	0.223
Living with HIV	14%	11%	3.28	0.194
Married/cohabitating	78%	64%	20.74***	0.000
Separated/divorced/widowed	21%	18%		
Single/never married	2%	18%		
None	14%	13%	0.87	0.832
Some primary	31%	29%		
Completed primary	20%	21%		
More than primary	35%	38%		
Age	53	42	6.50***	0.000
Number of children	5.1	4.7	0.76	0.448
Indegree	4.4	1.4	16.54***	0.000
Outdegree	2.0	1.8	1.80^	0.072
*Characteristics as reported by those nominating these individuals* #				
Highly trusted	28%	28%	0.05	0.829
Seen daily	46%	57%	17.30***	0.000
Friend	65%	60%	3.48^	0.062
Partner/spouse	5%	10%	12.94***	0.000
Other blood or family relation	18%	14%	3.64^	0.056
*Nomination order*				
Nominated 1st (most important)	11%	19%	16.42***	0.000
Nominated 2nd	21%	22%	0.15	0.702
Nominated 3rd	23%	18%	5.02*	0.025

Notes: Individuals with high authority scores were those in the top 10th percentile for authority score. ^ *p* < 0.10; *p < 0.05; **p < 0.01; ***p < 0.001.; ^#^ The characteristics of individuals with high authority were identified based on when they were nominated and averaged across all nominations they had received. The same was done for all other individuals in the sample, using the average from all nominations they received (e.g. trust)

**Table 7:** Differences in characteristics between individuals who are isolated versus non-isolated

	Isolated	Non-isolated	Χ^2^/ t-stat	*p*-value
Female	43%	56%	7.77**	0.005
Living with HIV	11%	12%	0.90	0.637
Married/cohabitating	52%	66%	25.63***	0.000
Separated/divorced/widowed	14%	18%		
Single/never married	34%	15%		
None	2%	13%	21.76***	0.000
Some primary	20%	29%		
Completed primary	21%	21%		
More than primary	56%	37%		
Age	35	43	−5.05***	0.000
Number of children	5.5	4.7	1.51	0.130

^ *p* < 0.10

* *p* < 0.05

** *p* < 0.01

*** *p* < 0.001

## Appendix

(See Table 7)
